# Peli1 controls the survival of dopaminergic neurons through modulating microglia-mediated neuroinflammation

**DOI:** 10.1038/s41598-019-44573-w

**Published:** 2019-05-29

**Authors:** Dongfang Dai, Jia Yuan, Yan Wang, Jing Xu, Chaoming Mao, Yichuan Xiao

**Affiliations:** 1 Department of Nuclear Medicine and Institute of Oncology, The Affiliated Hospital of Jiangsu University, Zhenjiang, Jiangsu 212001 China; 2 CAS Key Laboratory of Tissue Microenvironment and Tumor, Shanghai Institute of Nutrition and Health, Shanghai Institutes for Biological Sciences, University of Chinese Academy of Sciences, Chinese Academy of Sciences, Shanghai, 200031 China

**Keywords:** Inflammation, Parkinson's disease

## Abstract

Chronic neuroinflammation is known to contributes to the toxicity of neurodegeneration of Parkinson’s disease (PD). However, the molecular and cellular mechanisms controlling inflammatory responses in the central nervous system remain poorly understood. Here we found that a E3 ubiquitin ligase Peli1 is dramatically induced only in the substantia nigra (SN) of the human and mouse PD brains. The ablation of Peli1 significantly suppressed LPS-induced production of neurotoxic mediators and proinflammatory cytokines in SN and in primary microglia, whereas Peli1 is dispensable for the inflammatory responses in astrocyte. Accordingly, *Peli1* deficiency markedly inhibited neuron death induced by the conditioned medium from LPS-stimulated microglia. Mechanistical study suggested that Peli1 acts as a positive regulator of inflammatory response in microglia through activation of NF-κB and MAP kinase. Our results established Peli1 as a critical mediator in the regulation of microglial activation and neuroinflammation-induced death of dopaminergic neurons during PD pathogenesis, suggesting that targeting Peli1 may have therapeutic effect in neuroinflammation.

## Introduction

Parkinson’s disease (PD) is a neurodegenerative disease among people over 65 years old that causes the movement dysfunction, which is due to the death of tyrosine hydroxylase (TH)^+^ dopaminergic neurons in the substantia nigra (SN)^1,2^. Published studies have suggested that microglia-associated neuroinflammation is a common feature during PD pathogenesis^3–5^. Microglia are the innate immune cells that reside very early in the central nervous system (CNS), in which they surveil the local environment to maintain the CNS homeostasis under physiological condition. Under pathological conditions, microglia are over-activated and produce large amounts of neurotoxic mediators, such as interleukin (IL)-1β, and tumor necrosis factor (TNF), nitric oxide (NO)^5,6^. During PD pathogenesis, rather than exhibiting protective function, activated microglia are detrimental for the survival of dopaminergic neurons and contribute to the aggregation of disease progression^6–9^.

Microbial infections have long been recognized as an environmental trigger for the pathogenesis of PD^10^. Lipopolysaccharide (LPS), endotoxin from gram-negative bacteria, is a potent activator of microglia and damages dopaminergic neurons only in the presence of microglia^11,12^. In addition, intracranial infusion of LPS could induce the death of TH^+^ dopaminergic neurons in mice, and thus was commonly applied for the generation of animal models to mimic the PD symptoms^13^. Microglia were found to be the first responder and rapidly activated in LPS-induced inflammation, producing large amounts of neurotoxic factors. These factors like IL-1β can activate astrocyte to produce more neurotoxic mediators together with that produced by microglia, leading to the loss of dopaminergic neurons^14^. These studies collectively suggested that bacteria infection may be a critical mediator to promote the disease onset and progression of PD.

LPS is a ligand for toll-like receptor (TLR)4, which induces MyD88- and Trif-mediated activation of downstream signals, such as NF-κB, MAP kinases and interferon regulatory factors (IRFs), leading to the transcriptional activation of neurotoxic genes like *Il1b*, *Tnf*, *Nos2*, etc^15^. The Pellino (Peli) E3 ubiquitin ligases have been reported to play essential roles in the modulation of TLR, IL-1 receptor (IL-1R) and TNR receptor (TNRR) signaling in innate immune cells^16–19^, and in the regulation of adaptive immune cell activation^20,21^. Our recent studies have indicated that Peli1 protein is highly expressed in the brain and spinal cord, in which Peli1 is predominantly expressed in microglia, but not in other CNS-resident cells like neuron, astrocytes and oligodendrocytes. In such case, Peli1 functions as an important mediator for microglia activation and contribute to the pathogenesis of multiple sclerosis and viral encephalitis^19,22^.

Based on ours and others’ previous reports, the current study hypothesized that Peli1 could regulate the PD pathogenesis through modulating microglial inflammatory responses. Our experiments were first aimed to characterize Peli1 function in PD and secondly to decipher the mechanism how Peli1 regulate the survival of dopaminergic neurons during neuroinflammation.

## Results

### Peli1 is induced in the SN of human and mouse PD brain

**Figure 1:**
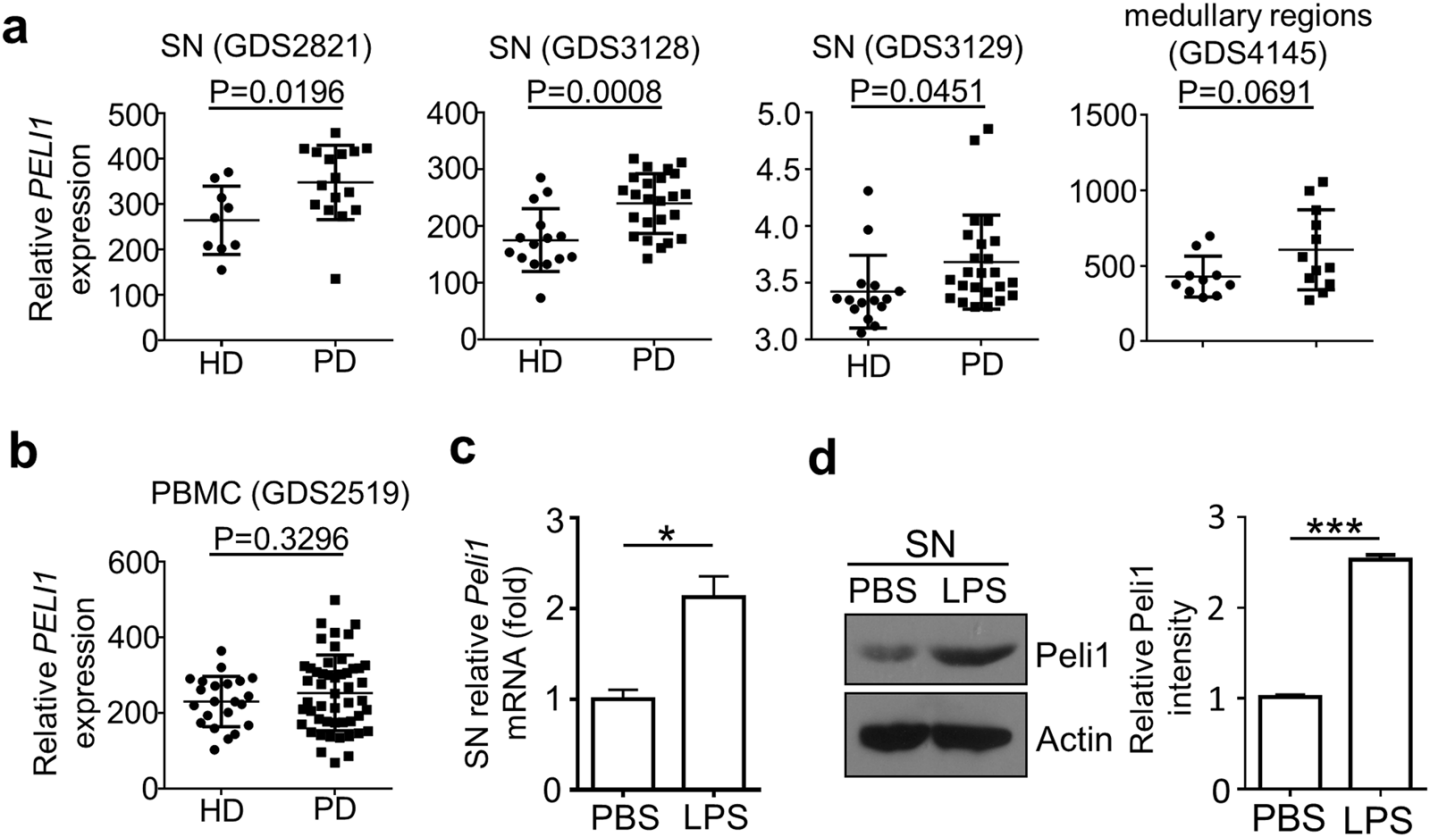
Peli1 is induced in the substantia nigra of human and mouse PD brains. (**a**,**b**) Normalized *PELI1* expression signals in peripheral blood mononuclear cells (PBMCs) (**a**) and substantia nigra (SN) (**b**) from health donors (HD) and Parkinson’s disease (PD) patients, the data were obtained from NCBI GEO dataset (GDS2821, GDS3128, GDS3129, GDS4145, GDS2519). Each dot in the graphs represent a value from one person. (**c**,**d**) QPCR and immunoblot analysis of relative mRNA (**c**) and protein (**d**) expression for Peli1 in the SN of WT and *Peli1*-KO mice that stereotaxically injected with 2 μl PBS or LPS (5 μg/ml). The immunoblot results are presented as Peli1 and Actin blot panels (d, left panel) and summary bar graph quantifying the relative Peli1 protein levels to Actin (**d**, right panel). Data with error bars represent mean ± SD. Each panel is representative of three independent experiment. *P < 0.05, ^***^P < 0.001 as determined by unpaired Student’s t test.

Recent evidences have suggested that not only CNS-resident cells, but also peripheral immune system, critically regulate the pathology of PD^23,24^. Since we previously demonstrated that Peli1 plays important roles in regulating the function of both CNS-resident microglia and peripheral immune cells^19,20,22^, so in order to examine the biological function of PELI1 during PD pathogenesis, we initially searched the public NCBI GEO database (GDS2519, GDS2821, GDS3128, GDS3129 and GDS4145) and examined the relative expression of *PELI1* in SN, medullary regions, and peripheral blood mononuclear cells (PBMCs) that isolated from health donors (HD) and PD patients. We found that not only one dataset (GDS2821, GDS3128 and GDS3129) suggested that *PELI1* expression is dramatically increased in the SN of PD patients as compared to that of HD (P = 0.0196, P = 0.0008, and P = 0.0451 Fig. 1a). Although there is no statistical difference, the data from GDS4145 suggested that the *PELI1* expression levels have an increased tendency in the medullary regions of PD patients as compared to that from HD (Fig. 1a). However, there is no obvious difference of *PELI1* mRNA expression in PBMCs between HD and PD patients (Fig. 1b). To confirm these observations, we stereotaxically injected LPS into the mouse SN to generate the inflammation-induced PD model, and examine the Peli1 induction. The result revealed that *Peli1* mRNA and its protein expression in SN is dramatically increased upon LPS priming as compared to that in PBS-injected control SN (Fig. 1c,d). These data collectively suggested that Peli1 may function directly in CNS-resident cells but not in the peripheral immune system to regulate PD pathogenesis.

### Peli1 deficiency protects against inflammation-induced TH^+^ neurons death

**Figure 2:**
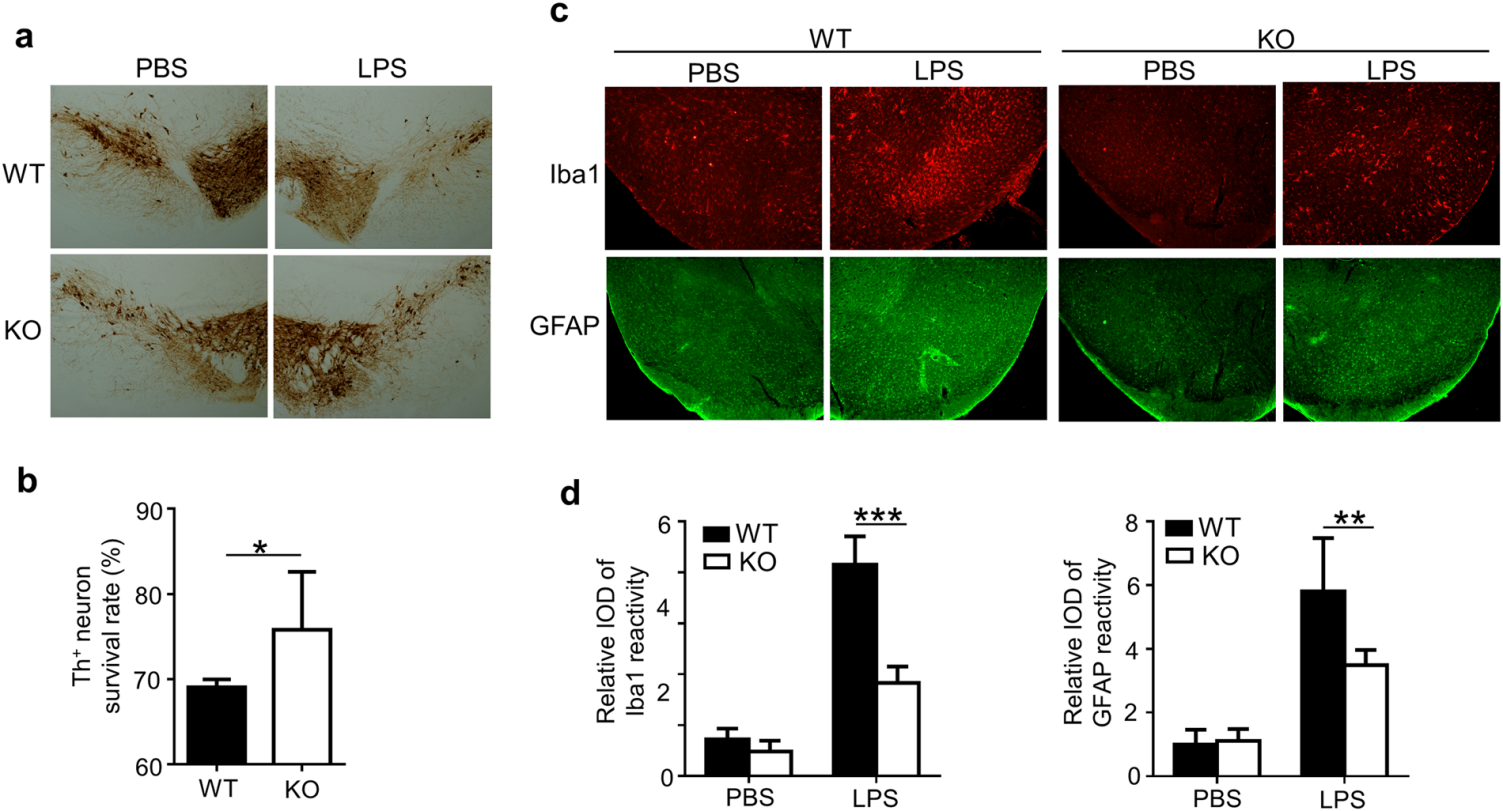
Peli1 deficiency suppresses inflammation-induced neuron death in substantia nigra. WT and *Peli1*-deficient mice (n = 4) were stereotaxically injected with 2 μl LPS (5 μg/ml) or equal volume of PBS into the substantia nigra (SN) of each mouse. (**a**,**b**) Immunohistochemical analysis of tyrosine hydroxylase (TH) in the ventral mesencephalon sections taken 7 days after PBS or LPS injection in WT and *Peli1*-KO mice. Data are showed as representative images (**a**) and summary bar graph showing the survival rates by calculating the ratios of TH^+^ neuron numbers of LPS-treated SN as compared to that of PBS-treated SN of the same mouse (**b**). (**c**,**d**) Immunofluorescent staining for Iba1 to indicate microglia, and GFAP to indicate astrocyte on the ventral mesencephalon of WT and *Peli1*-KO mice that injected with PBS or LPS. Data were presented as representative images (**c**) and summary bar graph showing the relative integrated optical density (IOD) of Iba1 and GFAP reactivity (**d**). Data with error bars represent mean ± SD. Each panel is representative of at least three independent experiment. *P < 0.05 as determined by unpaired Student’s t test (**b**) or two-way ANOVA with a Bonferroni post test (**d**).

To figure out whether Peli1 indeed modulate PD pathogenesis, we generated the PD model by injecting LPS into SN of WT and *Peli1*-KO mice, and then analyzed the survival condition of TH^+^ dopaminergic neurons by using the immunohistochemical staining of SN 7 days after LPS treatment. The data suggested that LPS injection induced a remarkable death of TH^+^ neurons in SN as reported. In contrast, *Peli1* deficiency markedly suppressed the loss and enhanced the survival rate of TH^+^ neurons in SN as compared to that in WT mice (Fig. 2a,b). Accordingly, we observed there are more accumulation of Iba1^+^ microglia and GFAP^+^ astrocytes in the SN of WT PD mice than that in KO PD mice (Fig. 2c,d), suggesting the suppressed activation of the glial cells in *Peli1*-KO mice during PD pathogenesis.

### Peli1 contributes to the enhanced inflammation in SN of PD mice

**Figure 3:**
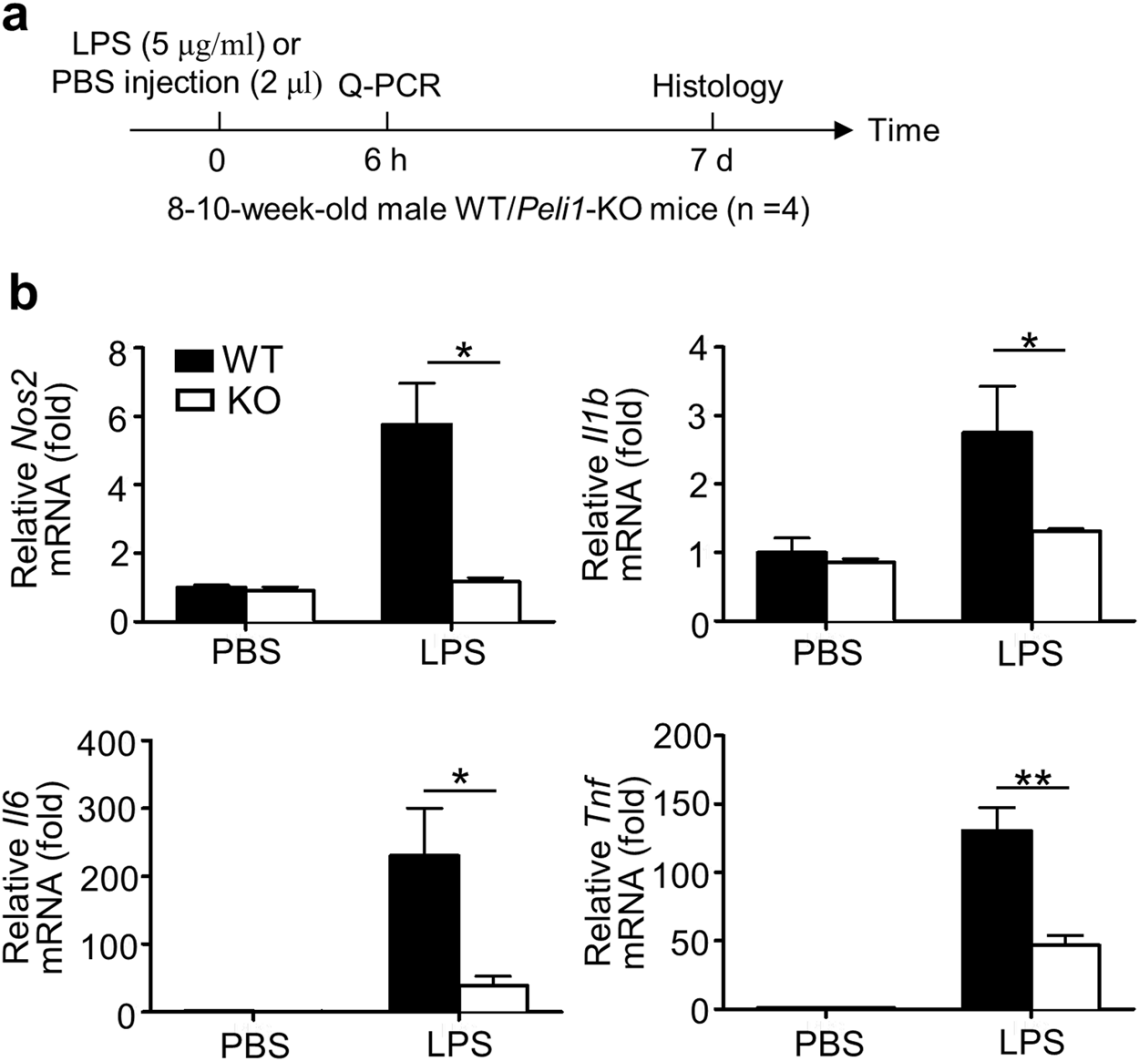
Peli1 deficiency suppresses LPS-induced neuroinflammation in substantia nigra. (**a**) Experimental diagram showing the procedure of LPS-induced neuroinflammation in SN and the time points when to collect tissue sample for QPCR or histological analysis. (**b**) QPCR analysis of relative mRNA expression for *Nos2* and the indicated proinflammatory genes in the SN of WT and *Peli1*-KO mice that stereotaxically injected with 2 μl PBS or LPS (5 μg/ml). The gene expression were normalized to a reference gene *Actb* (encoding β-actin) and showed as the relative value to *Actb* in bar graphs. Data with error bars represent mean ± SD. Each panel is representative of at least three independent experiment. **P < 0.01 as determined by two-way ANOVA with a Bonferroni post test (**b**).

To confirm that Peli1 mediates the enhanced inflammation in SN during PD pathogenesis, we examined the expression of proinflammatory genes in SN (Fig. 3a). In consistent with the immunofluorescence data, Peli1 deficiency markedly inhibited LPS-induced expression of *Nos2* and proinflammatory genes (*Il1b*, *Il6*, *Tnf*) in SN as compared to that injected with PBS (Fig. 3b), suggesting that Peli1 served as a positive regulator of neuroinflammation in PD pathology.

### Peli1 mediates LPS-induce production of neurotoxic factors in microglia

**Figure 4:**
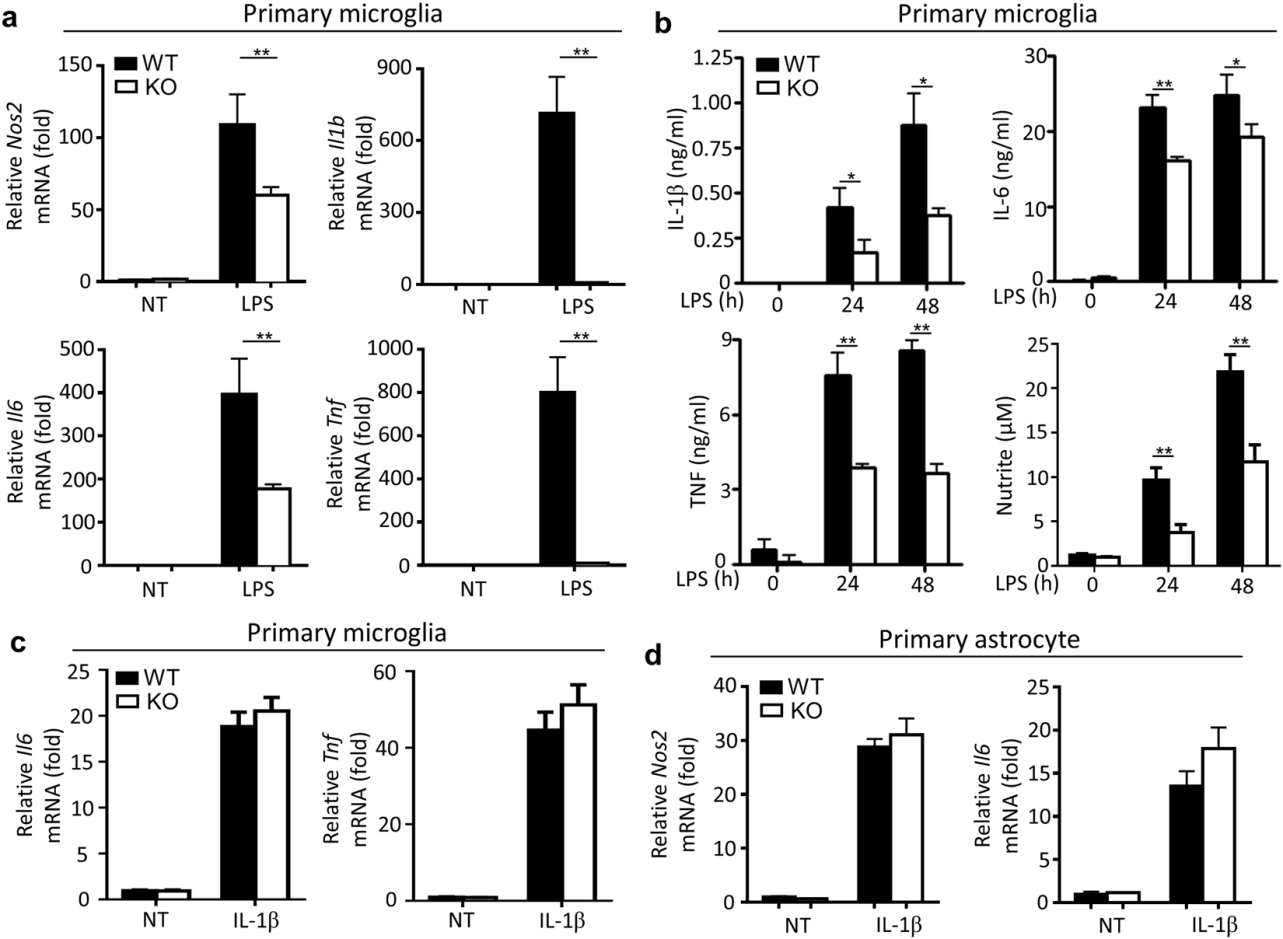
Peli1 mediates LPS-induce inflammatory responses in microglia. (**a**) QPCR analysis of relative mRNA expression for the indicated inflammatory genes in the primary cultured microglia isolated from WT and *Peli1*-KO mice, left untreated (NT) or stimulated with LPS (100 ng/ml). (**b**) ELISA of IL-1β, IL-6, TNF and nitrites in the supernatant of WT and *Peli1*-KO microglia cultures that left untreated (NT) or stimulated with LPS (100 ng/ml) at the indicated time points. (**c**,**d**) QPCR analysis of relative mRNA expression for the indicated inflammatory genes in the primary cultured microglia (**c**) or astrocytes (**d**) isolated from WT and *Peli1*-KO mice, left untreated (NT) or stimulated with IL-1β (20 ng/ml). The gene expression were normalized to a reference gene *Actb* (encoding β-actin) and showed as the relative value to *Actb* in bar graphs. Data with error bars represent mean ± SD. Each panel is representative of at least three independent experiment. *P < 0.05, **P < 0.01 as determined by two-way ANOVA with a Bonferroni post test.

It is reported that microglia are initially activated and responsible for the neurotoxicity of LPS-mediated neuroinflammation in SN^25^, so we tested the LPS-mediated gene induction in mouse primary microglia. Expectedly, we found that LPS-induced expression of *Nos2* and proinflammatory genes that encoding IL-1β, IL-6 and TNF, were significantly inhibited in *Peli1*-deficient microglia (Fig. 4a). Accordingly, the loss of Peli1 in microglia significantly suppressed the secretion of these proinflammatory cytokines and neurotoxic nitrites in the microglial cell culture supernatant (Fig. 4b). Therefore, these results suggested that Peli1 mediates the activation and production of neurotoxic factors in microglia during PD pathogenesis.

### Peli1 does not affect the inflammatory responses in astrocytes

Published studies showed that astrocytes have also been well-characterized to function as a key modulator of neuroinflammation during neurodegeneration^26,27^. In this model, astrocyte can be activated by the IL-1β and/or TNF that produced by activated microglia, and together with microglia, synergistically promote the neurotoxic effect^25^. Although Peli1 is known as a positive regulator of IL-1R signaling in innate immune cells^16–18^, we found that IL-1β-induced inflammatory responses are not affected in *Peli1*-deficient microglia and astrocyte as compared to WT cells (Fig. 4c,d). In addition, we have previously demonstrated Peli1 is dispensable for the inflammatory responses induced by TNF in astrocytes^19^. Together, these results demonstrated that the suppression of LPS-induced neuroinflammation in SN of *Peli1*-deficient mice is due to the defective activation of microglia, but not astrocyte.

### Peli1 deficiency inhibits microglia-mediated neuron death

**Figure 5:**
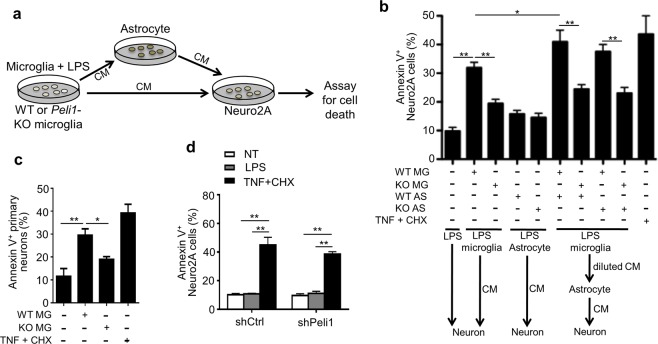
Peli1 deficiency inhibited microglia-mediated neuron death. (**a**) Scheme of conditioned media (CM) and cell death assay by using WT and/or *Peli1*-KO microglia or astrocytes. (**b**,**c**) CMs were harvested from WT and *Peli1*-KO microglia that were stimulated with LPS (100 ng/ml) for 24 h, and then used for the test of viability of Neuro2A cells (**b**) or primary neurons (**c**). For sequential CM assay, CMs harvested from microglia were cultured with WT and *Peli1*-KO astrocytes for 24 h. Then, CMs were harvested and tested for effect on viability of Neuro2A cells. Neuro2A cells that treated with TNF (20 ng/ml) plus cycloheximide (CHX) were used as a positive control. (**d**) Effect of *Peli1* knockdown in Neuro2A cells on sensitivity to TNF (20 ng/ml) plus CHX induced apoptosis assessed by flow cytometry assay, showing the frequencies of Annexin V^+^ cells in bar graph. Data with error bars represent mean ± SD. Each panel is representative of at least three independent experiment. *P < 0.05, **P < 0.01 as determined by one-way ANOVA with a Tukey’s post test (**b**–**d**).

To confirm that Peli1-mediated microglial activation is responsible for the neuron death during LPS-induced SN inflammation, we examined the neurotoxicity to Neuro2A cells by using different conditioned medium (CM) that isolated from LPS-stimulated primary cultured WT or *Peli1*-deficient microglia or astrocyte (Fig. 5a). We also used TNF plus cycloheximide (CHX) stimulation as a positive control, which can induce Neuro2A cell death. As expected, WT microglia-derived CM significantly promoted the apoptosis of Neuro2A cells, whereas CM from *Peli1*-deficient microglia dramatically inhibited Neuro2A cell death. However, CM of astrocytes from both WT and *Peli1*-deficient mice didn’t affect the survival of Neuro2A cells, and there is no significant difference of these two groups. Interestingly, WT microglia-derived CM significantly promoted the production of neurotoxic factors of both WT and *Peli1*-deficient astrocytes. This effect was markedly inhibited when using the CM from *Peli1*-deficient microglia (Fig. 5b). More interestingly, CM from WT primary microglia also induced significant cell death of primary neurons, but *Peli1* deficiency in microglia almost abolished the toxic effect on primary neurons that induced by microglia-derived inflammatory mediators (Fig. 5c**)**.

To exclude the possibility that Peli1 in neurons may directly regulate the inflammation-induced cell death, we knocked down *Peli1* gene expression in Neuro2A cells and stimulated with TNF plus CHX, a positive inducer of cell death. The results showed in Fig. 5d indicated that *Peli1* knockdown in Neuro2A cells does not affect neuron cell death induced by TNF plus CHX. These results further confirmed that Peli1-mediated activation and production of neurotoxic mediators in microglia contribute to the neuron death during LPS-induced SN inflammation.

### Peli1 mediates NF-κB and MAP kinase activation in microglia

**Figure 6:**
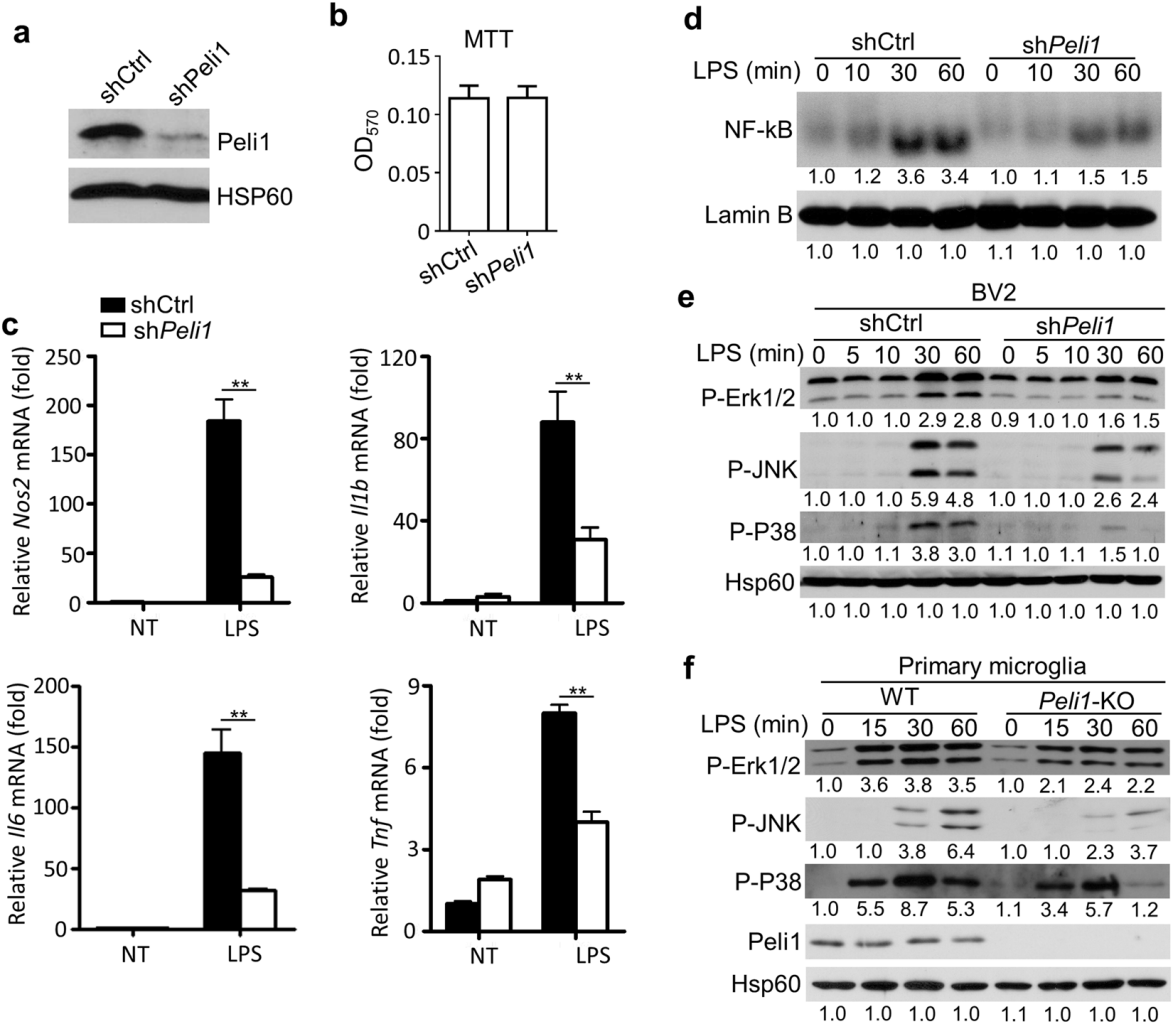
Peli1 mediated NF-κB and MAP kinase activation in microglia. (**a**) Immunoblot analysis of Peli1 showing the knockdown efficiency in murine BV2 microglial cells, Hsp60 immunoblot was used as a loading control. (**b**) MTT analysis examining the survival of control and Peli1-knockdown BV2 cells. (**c**) QPCR analysis of relative mRNA expression for the indicated inflammatory genes in the BV2 cells that infected with lentivirus encoding control (shCtrl) or *Peli1* shRNA (sh*Peli1*), left untreated (NT) or stimulated with LPS (100 ng/ml). The genes’ expression were normalized to a reference gene *Actb* (encoding β-actin) and showed as the relative value to *Actb* in bar graphs. (**d**) Electrophoretic mobility-shift assay (EMSA) of NF-κB in nuclear extracts of control or *Peli1-*knockdown BV2 cells left unstimulated or stimulated with LPS (100 ng/ml) at the indicated time points. Lamin B immunoblot was used as a loading control. (**e**,**f**) Immunoblot analysis of phosphorylated (P-) MAP kinases in whole-cell lysates of control or *Peli1-*knockdown BV2 cells (**e**) or in primary microglia (**f**) that left unstimulated or stimulated with LPS (100 ng/ml) at the indicated time points. The relative protein levels were quantified by ImageJ and the values were presented below each blot. Data with error bars represent mean ± SD. Each panel is representative of at least three independent experiment. **P < 0.01 as determined by unpaired Student’s t test (**b**) or two-way ANOVA with a Bonferroni post test (**c**).

To dissect the molecular mechanisms of Peli1-mediated microglial activation, we reduced Peli1 expression in murine BV2 microglia cells (Fig. 6a), and *Peli1* knockdown does not affect the growth and survival of BV2 cells (Fig. 6b). We next investigated the function of Peli1 in modulating LPS-induced gene expression in Peli1-knockdown BV2 cells. The results revealed that the expression of *Nos2* and proinflammatory cytokine genes were significantly inhibited in *Peli1*-knockdown BV2 cells in response to LPS stimulation (Fig. 6c), which confirmed the phenotype we discovered in primary microglia. Since NF-κB and MAP kniase activation play important role in LPS-mediated inflammation, we next tested the activation status of these signal pathways in BV2 cells. The results indicated *Peli1* knockdown in BV2 cells significantly inhibited NF-κB activation by EMSA analysis, and MAP kinase activation by immunoblot analysis (Fig. 6d–f), which were consistent with our previously published data obtained from primary microglia. Taken together, Peli1 mediated LPS-induced activation of NF-κB and MAP kniase, and thus elevated the activation neurotoxic gene expression in microglia.

## Discussion

The specific loss of dopaminergic neurons in the SN is the hallmark of PD^10,28^. Until now, the detailed mechanism controlling dopaminergic neuronal cell death during PD pathogenesis are not fully understood. However, published studies have suggested that the local inflammation in SN may be function as an active contributor to promote the pathology of both human and mouse PD^29–31^. Therefore, an improved understanding of the mediators that regulate CNS inflammatory responses during PD pathogenesis will promote the understanding of the pathogenic mechanism of PD and thus help develop new therapeutic strategies. Here, we found that the E3 ubiquitin ligase Peli1 function as an important mediator to regulates the pathogenesis of PD. Loss of Peli1 significantly protected neurons from inflammation-induced neurotoxicity.

Microglial activation has been recognized as a critical event that initiates inflammatory responses in the pathogenesis of PD^14,25^. In the present study, we found that deficiency of Peli1 in primary cultured microglia dramatically suppressed LPS-induced secretion of neurotoxic mediators, leading to promoted neuron cell survival. However, Peli1 deficiency does not affect astrocyte activation by IL-1β stimulation, which is consistent with previous report that Peli1 is dispensable for the IL-1R signaling pathway in mouse embryo fibroblasts (MEF)^32^. In addition, LPS-mediated microglial activation and production of neurotoxic factor are sufficient to induce neuron death. *Peli1* deficiency in microglia obviously inhibited this inflammation-induced cell death. More interestingly, LPS-induced pro-inflammatory cytokines by microglia further exhibit a paracrine function to activate astrocytes, which increase the production of neurotoxic factors, leading to exaggerated neurotoxic effect. Consistent with the gene expression results, *Peli1* deficiency in astrocyte does not affect neuron death by astrocyte conditioned medium that stimulated with the same microglial conditioned medium. Collectively, our data further confirmed that microglial activation is a critical initiator of inflammation in PD pathogenesis.

The microglia-specific function of Peli1 may be attribute to its expression pattern in different glial cells. Our previous data have shown that astrocyte express three Peli family members (Peli1/2/3) at comparable levels^19^. Given the extremely high levels of sequence identity of the different Peli proteins, it is very likely that they have functional redundancies in IL-1R signaling functions in astrocyte. Due to the predominant expression of Peli1 in microglia, knockdown of Peli1 in BV2 cells not only affected the TRIF-mediated TLR signaling in NF-κB pathway, but also impaired the MyD88-dependent TLR signaling in the activation of MAPKs. These results are consistent with our previously findings in *Peli1*-deficient innate immune cells^32^ and primary microglia^19^.

Although no evidences indicated that LPS or bacterial infection directly involve in the human PD pathogenesis, very little LPS has been found in the brains under normal physiological condition^33^, suggesting a possibility that the entered LPS may initiate the neuroinflammation. Accumulating evidences suggested that aging increase the generation of endogenous TLR ligands, which may promote the disease initiation or progression^34,35^. In fact, repeated intraperitoneal injection of LPS exacerbates motor axon degeneration in the mouse system^36,37^, suggesting LPS or bacteria infection may be a risk factor to promote the neuroinflammation and thus induce the death of dopamine neurons. The present findings further confirmed that modulation of LPS-induced TLR inflammatory signaling by Peli1 in microglia indeed controlled the pathogenesis of PD.

In conclusion, we provided the evidences that Peli1 is induced in the SN of human and mouse PD brain, in which Peli1 facilitates microglial over-activation through NF-κB and MAP kinases. The activated microglia that produced amounts of neurotoxic factors, together with the inflammatory mediators produced by activated astrocytes, contributed to inflammation-induced death of dopaminergic neurons. Therefore, targeting Peli1 may suppress expression of neurotoxins, and thus have therapeutic effect in neuroinflammation and related neurological diseases.

## Methods

### Mice

*Peli1*-deficient mice (on the C57BL/6 background) were obtained as described previously^21^. Briefly, *Peli1*^+/−^ heterozygous mice were bred to generate age-matched *Peli1*^−/−^ (*Peli1*-KO) and *Peli1*^+/+^ (WT) mice. Mice were maintained in a specific pathogen-free facility, and all animal experiments were in accordance with protocols approved by the Institutional Animal Care and Use Committee of Shanghai Institutes for Biological Sciences, Chinese Academy of Sciences.

### Antibodies and reagents

Antibodies for phospho-JNK (4668) and phospho-p38 (9215) were purchased from Cell Signaling Technology Inc. The anti-phospho-ERK1/2 (sc-7383), Peli1 (sc-271065), Hsp60 (sc-13115), iNOS (sc-7271) and Lamin B (sc-6216) antibodies were from Santa Cruz. The anti-TH antibody (MAB318) was from Chemicon, anti-Iba1 antibody (019-19741) was from WAKO, and anti-GFAP antibody (G3893) were from Sigma-Aldrich. LPS (L3129) was from Sigma-Aldrich. The cycloheximide (CHX) (HY12320) was from MCE. Murine IL-1β (211-11B) and TNF (315-01 A) were from Peprotech.

### *PELI1* gene expression analysis of human samples

To analyze the relative *PELI1* expression in human samples, we extracted the data from NCBI GEO database (GDS2821, GDS3128, GDS3129, GDS4145, GDS2519), and compared the expression of *PELI1* in the PBMCs or SN from health donors and PD patients.

### LPS-induced PD mouse model

The induction of PD mouse model was as described previously^25^. Briefly, WT and *Peli1*-defiicient 8-10-week-old male mice (n = 4–5 mice/group) were anesthetized and then immobilized in a stereotaxic apparatus. Two microliters of LPS (5 μg/ml) or equal volume of PBS were stereotaxically injected with into the SN (AP, −3.3 mm; ML, ±1.2 mm; and DV, −4.6 mm) over a 5-minute period by using a stainless-steel syringe (Hamilton). Mice were euthanized 6 h after the stereotaxic injection, and the injected SN was dissected under a dissection microscope and the tissue was processed for quantitative PCR. Seven days after the injection, the mice were sacrificed, and the brain slices were collected for immunohistochemistry analysis.

### Immunohistochemistry (IHC) and immunofluorescence (IF)

The IHC or IF staining was as described previously^25,26^. Experimental mice were anesthetized and perfused transcardially with PBS followed by 4% paraformaldehyde. Brain samples were postfixed with 4% paraformaldehyde overnight and equilibrated in 30% sucrose. Coronal sections of 10 μm were prepared with a sliding microtome, and were then incubated with primary antibodies: rabbit anti-tyrosine hydroxylase pAb (1:500; Chemicon); rabbit anti-Iba1 pAb (1:500; WAKO); rabbit anti-GFAP pAb (1:800; DAKO); mouse anti-GFAP mAb (1:1,000, Sigma-Aldrich). The brain slices were then incubated with the horseradish peroxidase (HRP)- or fluorescence-conjugated secondary antibodies. The peroxidase activity of immune complexes was revealed with a DAB kit according to the manufacturer’s instruction (Beyotime, P0203). Sections were imaged using either a cooled CCD (DP72, Olympus) on a microscope (BX51; Olympus).

### Cell quantification

The number of tyrosine-hydroxylase (TH)-positive cells was quantified in adult *Peli1*-KO and their littermates in brain cryosections with typical morphology of the substantia nigra as previously described^26^. In brief, four series of cryosections were collected and every fourth section (10 μm) was used for quantification of TH-positive neurons. The average intensities of GFAP or Iba1 were calculated using ImageJ in the substantia nigra.

### Primary glial cell culture

Mixed glial cultures were prepared from neonatal mice that age around 1–2 days as described previously^26^. In brief, after removing the meninges, the neonatal brains were dissociated by 0.25% trypsin, filtered with a 40-μm mesh, and the dissociated cells were plated in 10 cm dish in DMEM/ F12 medium containing 10% FBS, penicillin and streptomycin at 37 °C in humidified 5% CO_2_/95% air. Culture media were changed twice a week. The mixed glial cells were reached to 90% confluence at around day 9, were then re-plated after trypsinization. At day 20 *in vitro*, cultures were mildly trypsinized with trypsin solution (0.07% trypsin in DMEM/Ham’s F12) at 37 °C for 15–20 min. Floating cells (astrocytes and dead cells) were removed by rinsing cultures with D-Hanks’ solution. The resulting enriched microglial cultures were maintained in DMEM/Ham’s F12 complete medium containing 10% FBS, penicillin and streptomycin until use. The purity of the microglia was >97% as determined by flow cytometry to measure the percentage of CD11b^+^ cells.

### Primary neuron culture

The primary mouse neurons were prepared by following our previous protocol^19^. Briefly, we dissociated the cortex of newborn mouse brains in 0.25% trypsin, filtered the cell suspension with a 40 μm mesh and cultured the cells in Neurobasal medium (Invitrogen) supplemented with B-27 (Invitrogen), 100 U/ml penicillin and 100 mg/ml streptomycin. The culture medium was changed every 3 d for 2 weeks. The collected primary neurons were then applied for the survival assay by using the CM from LPS-stimulated primary microglia.

### Quantitative RT-PCR

Brain tissues or cell samples were homogenized in Trizol reagent^26^. cDNA was synthesized from 1 μg of extracted total RNA using M-MLV Reverse Transcriptase kit (Takara) according to the manufacturer’s instructions. Quantitative PCR was performed with SYBR-Green premix ExTaq (Roche) and detected by a Real Time PCR System by using gene-specific primer sets (Table 1). The relative genes’ expression was assessed in triplicate, normalized to a reference gene *Actb* (encoding β-actin) and determined based on 2^−∆∆Ct^ method^38^.

**Table 1:** Primers used for real-time quantitative PCR.

Genes	Forward primers	Reverse primers
*Il6*	CACAGAGGATACCACTCCCAACA	TCCACGATTTCCCAGAGAACA
*Tnf*	CATCTTCTCAAAATTCGAGTGACAA	CCAGCTGCTCCTCCACTTG
*Il1b*	AAGCCTCGTGCTGTCGGACC	TGAGGCCCAAGGCCACAGGT
*Nos2*	GTGGTGACAAGCACATTTGG	AAGGCCAAACACAGCATACC

### Measurement of the inflammatory cytokines and nitrite

The concentrations of mouse IL-1β, TNF and IL-6 in the supernatants of primary microglial cultures that with or without stimulation were measured through enzyme-linked immunosorbent assay (ELISA). Briefly, the 96-well flat plates were coated with the capture antibodies for IL-1β (eBioscience, 14-7012-81), TNF (eBioscience, 14-7325-85) or IL-6 (eBioscience, 14-7061-85) at 4 °C overnight. The microplates were blocked with 3% BSA, incubated with the supernatants at room temperature for 2 h, and followed by incubation with biotinylated detection antibodies for IL-1β (eBioscience, 13-7112-81), TNF (eBioscience, 13-7341-85) or IL-6 (eBioscience, 13-7062-85). Then HRP-conjugated avidin (eBioscience, 18-4100-51) were added into each well for 30 min at room temperature, visualized by TMB solution (eBioscience, 00-4201-56) and quantified through a TECAN ELISA reader. The nitrite concentration in the supernatants were examined by using a NO assay kit (Beyotime, S0021) according to the manufacturer’s instruction.

### Immunoblot (IB) and electrophoresis mobility shift assays (EMSA)

IB analysis were performed as previously described^19^. Primary microglia were stimulated with 100 ng/ml LPS for the indicated time period and lysed in RIPA buffer. The whole cell extracts were separated via 8.25% SDS-PAGE, transferred to PVDF membranes, blocked and subject to IB analysis. Nuclear extract preparation and EMSAs were carried out as described previously^19^.

### Statistical analysis

Statistical analysis was performed as previously described^39^. Differences between groups were established using a Student’s unpaired t test (for two groups), a one-way ANOVA with a Tukey’s post test for multiple groups’ comparisons, or a two-way ANOVA with a Bonferroni post test when comparing groups with multiple variables. Data are presented as means ± SD. A *P*-value less than 0.05 is considered statistically significant. Statistic details are indicated in the respective figure legends.

### Ethics approval and consent to participate

All study surgical procedures and experiment protocols were performed in accordance with protocols approved by the Institutional Animal Care and Use Committee of Shanghai Institutes for Biological Sciences, Chinese Academy of Sciences (Shanghai, China).
